# GWAS Reveal Targets in Vessel Wall Pathways to Treat Coronary Artery Disease

**DOI:** 10.3389/fcvm.2018.00072

**Published:** 2018-06-25

**Authors:** Adam W. Turner, Doris Wong, Caitlin N. Dreisbach, Clint L. Miller

**Affiliations:** ^1^Center for Public Health Genomics, University of Virginia, Charlottesville, VA, United States; ^2^Department of Biochemistry and Molecular Genetics, University of Virginia, Charlottesville, VA, United States; ^3^Data Science Institute, University of Virginia, Charlottesville, VA, United States; ^4^Department of Public Health Sciences, University of Virginia, Charlottesville, VA, United States

**Keywords:** genome-wide association study (GWAS), coronary artery disease (CAD), drug targets, smooth muscle cells, vascular wall

## Abstract

Coronary artery disease (CAD) is the leading cause of mortality worldwide and poses a considerable public health burden. Recent genome-wide association studies (GWAS) have revealed >100 genetic loci associated with CAD susceptibility in humans. While a number of these loci harbor gene targets of currently approved therapies, such as statins and PCSK9 inhibitors, the majority of the annotated genes at these loci encode for proteins involved in vessel wall function with no known drugs available. Importantly many of the associated genes linked to vascular (smooth muscle, endothelial, and macrophage) cell processes are now organized into distinct functional pathways, e.g., vasodilation, growth factor responses, extracellular matrix and plaque remodeling, and inflammation. In this mini-review, we highlight the most recently identified loci that have predicted roles in the vessel wall and provide genetic context for pre-existing therapies as well as new drug targets informed from GWAS. With the development of new modalities to target these pathways, (e.g., antisense oligonucleotides, CRISPR/Cas9, and RNA interference) as well as the computational frameworks to prioritize or reposition therapeutics, there is great opportunity to close the gap from initial genetic discovery to clinical translation for many patients affected by this common disease.

## Introduction

Coronary artery disease (CAD) is a maladaptive inflammatory disease of the coronary artery vessel wall that remains one of the leading causes of death worldwide. It involves numerous cell types (smooth muscle cells, endothelial cells, and macrophages) and often manifests in myocardial infarction. Development of CAD is due to a combination of genetic and environmental factors. Early twin studies indicated CAD heritability was ~40-60% (1, 2). Linkage and family-based studies identified genes with now well-established roles in disease pathogenesis, such as the LDL receptor (LDLR) (3), apolipoprotein B (apoB) (4), and proprotein convertase subtilisin/kexin type 9 (PCSK9) (5).

In 2007 the first genome-wide association studies (GWAS) of CAD published the association of the 9p21 locus with both CAD and myocardial infarction (MI) (6–8). The 9p21 locus remains the most robust locus in the genome with respect to CAD association. Many more CAD loci have been discovered in subsequent GWAS over the past decade, leading to the formation of the CARDIoGRAM (9) and C4D (10) consortia and resulting meta-analyses (11–15). The most recent GWAS meta-analysis for CAD has ~300,000 combined cases and controls and identified almost 100 independent loci reaching genome-wide significance (*p* < 5 × 10^−8^), and over 300 loci significant at a 5% false discovery rate.

**Figure 1:**
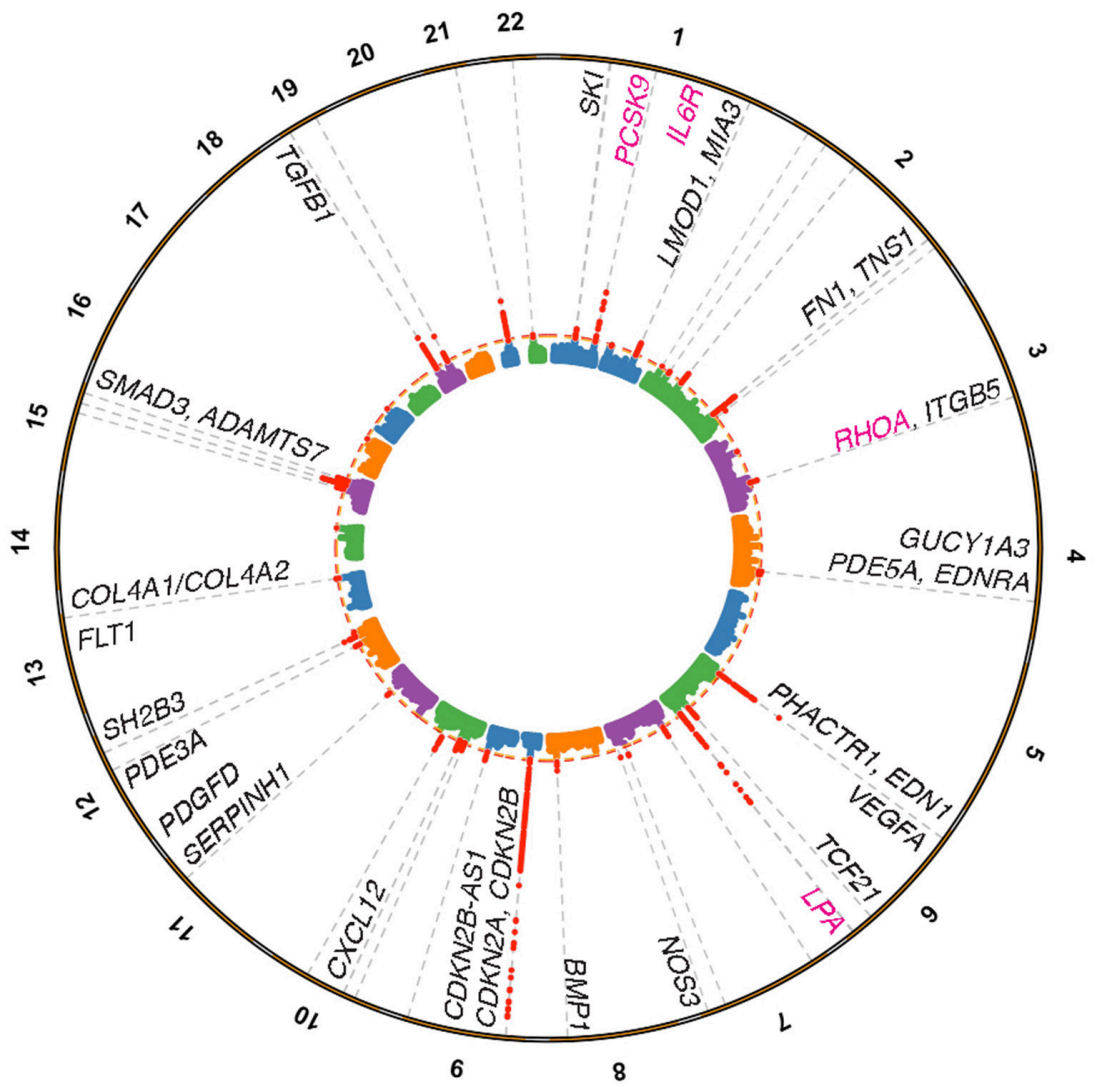
Coronary artery disease loci harboring genes linked to vessel wall functions. Manhattan plot depicting genome-wide significant loci identified from the Nelson et al. (15) meta-analysis for CAD based on a 5% false discovery rate using the pilot UK Biobank data. Loci were annotated through a combination of gene expression, epigenomic features, eQTL, and literature based searches. Vessel wall gene symbols are shown above associations in red, with dotted orange line representing *P* = 5.0 × 10^−8^. Pink gene symbols represent loci with either approved or evaluated treatments for CAD.

Despite the discovery of many new loci associated with CAD, the current challenges are to validate the causal genes and pathways at CAD loci and to translate this knowledge into new therapies. In this mini-review, we highlight recent GWAS identified non-lipid genes and pathways (with an emphasis on vessel wall pathways) that have the potential to accelerate new treatments for CAD (Figure 1). In addition, we provide some genetic perspective on currently approved and future therapies, as well as the use of genetic risk scores (GRS) to identify high risk patients who may require these novel treatments to augment traditional lipid-lowering therapy.

## CAD GWAS genes and pathways

### Vessel wall signaling

Once atherogenic lipoproteins have crossed the endothelium and are taken up by macrophage-derived foam cells, there is a subsequent cascade of complex signaling events in the vessel wall. This involves a tightly orchestrated interplay of vascular smooth muscle cells, endothelial cells, macrophages, cytokines, and extracellular matrix proteins. Reactome pathway gene-set enrichment analysis carried out by the CARDIoGRAM consortium indicated that CAD genes were enriched for pathways involved in NO/cGMP signaling, TGFβ/SMAD signaling, PDGF signaling, extracellular matrix (ECM) integrity/organization, and innate immunity (16). Further integrative analyses of CARDIoGRAM summary data, tissue-specific regulatory networks and gene expression data have revealed interactions across CAD-relevant pathways as well as potential druggable targets such as LUM and STAT3, which serve as key regulators of vessel wall biology (17). Assuming that the genes in these pathways are the most likely causal genes at the associated loci, these results argue that vascular wall pathways have comparable associations to the well-established lipid and lipoprotein mediated pathways (16). In fact up to 75% of the 95 CAD loci (15) appear to be associated independently of classical risk factors. This observation suggests that these risk factors are intrinsic to dysregulated processes in the vessel wall.

### NO/cGMP signaling

NO/cGMP signaling is fundamental to diverse cardiovascular physiological responses and emerging evidence suggests that activation of this pathway is defective in the setting of atherosclerosis and CAD. Nitric oxide (NO) is an important gas that is synthesized by endothelial nitric oxide synthase (eNOS), which upon activation results in paracrine signaling through the myoendothelial junction to smooth muscle cells, subsequent activation of soluble guanylate cyclase, cGMP production, and cGMP-dependent protein kinase (protein kinase G; PKG) mediated phosphorylation of downstream targets involved in vasodilation. The 1000 Genomes based CARDIoGRAMplusC4D (12) and recent UK Biobank-CARDIoGRAMplusC4D meta-analysis (15) identified an association for rs3918226 at *NOS3*, the gene which encodes eNOS, implicating a role in endothelial dysfunction. An intronic variant rs7692387 in *GUCY1A3*, encoding the alpha1-subunit of sGC, was associated with CAD (11), while another variant rs13139571 was associated with systolic (SBP) and diastolic (DBP) blood pressure (18). Recent functional studies identified a mechanism by which the non-risk allele at rs7692387 preferentially binds the ZEB1 transcription factor leading to increased *GUCY1A3* expression and sGC levels, which correlated with reduced atherosclerosis severity in mice (19). Other members of this pathway that have been linked to CAD include recently identified *PDE5A* (rs7678555) (15) and *PDE3A*, previously associated at 5% FDR (11), suggesting alterations in vascular wall signaling could be rescued with existing therapies (e.g., sildenafil, ciloztasol).

### TGFβ and PDGF signaling

The CARDIoGRAM GWAS studies have implicated several components of the transforming growth factor beta (TGFβ) signaling pathway in CAD. Activated TGFβ receptor I phosphorylates receptor-regulated SMAD proteins (SMAD3 or SMAD2). These are transcriptional mediators of TGFβ signaling that along with SMAD4 translocate to the nucleus to regulate transcription of TGFβ target genes. The *TGF*β1 and *SMAD3* genes are both associated with CAD in addition to bone morphogenic protein 1 (*BMP1*), a member of the TGF beta superfamily (20). Mechanistic studies have implicated a functional intronic SNP in *SMAD3* (rs17293632) that disrupts binding of the AP-1 transcription factor complex underlying this association (21, 22). The genetic association of rs36096196 at the *SKI* locus suggests a role for SKI, a co-repressor of SMAD3/SMAD2 signaling in CAD (23).

The rs150512726 SNP [proxy for the recently reported SNP rs142695226 (15)] results in a 3 amino acid deletion in the integrin beta 5 (ITGB5) protein. ITGB5 has been shown to play a role in activation of the latent TGFβ precursor protein outside the cell (24). The TGFβ pathway also regulates gene expression at the 9p21 locus. SNPs at this locus disrupt TEAD factor binding and the TEAD3-dependent TGF beta induction of p16 in human aortic smooth muscle cells (25).

The CARDIoGRAM studies have also identified SNPs at the platelet-derived growth factor D (*PDGFD*) locus associated with CAD at genome-wide significance. This PDGF mediated pathway may involve many other risk-associated genes. Preliminary work by our group has provided evidence of cross-talk with smooth muscle cell enriched pathways using genome-wide profiling of these cells. For example, the expression of *TCF21*, a transcription factor which determines the fate of epicardial progenitor cells during development, is increased in individuals carrying the risk alleles, rs121902987 and rs12524865 (26). Its expression was positively regulated by PDGF-BB-PDGFRB stimulation in human coronary artery smooth muscle cells (26). *TCF21* dysregulation likely increases CAD risk by altering coronary artery smooth muscle cell responses to vascular injury during plaque remodeling (27, 28). Another vessel wall gene, *LMOD1*, an actin filament nucleator, was shown to be downregulated in vascular smooth muscle cells in response to PDGF treatment and serves as a potent marker of smooth muscle cell phenotypic modulation (29).

### Extracellular matrix remodeling pathways

The CARDIoGRAM consortium has highlighted numerous extracellular matrix and basement membrane genes involved in the pathogenesis of atherosclerosis, including *COL4A1/COL4A2, ITGB5*, and *FN1*. A *COL4A2* variant, rs4773144, was associated with both *COL4A1* and *COL4A2* expression, as well as smooth muscle cell survival, and plaque stability (30). The authors suggest type IV collagen levels affect SMC proliferation, migration, extracellular matrix remodeling, apoptosis, and infiltration of immune cells through plaque remodeling. The CAD locus *MIA3* is involved in the endoplasmic reticulum export of large cargo such as pre-chylomicrons/VLDL (31) and collagens (including Col4a1 and Col4a2 in mice) (32). The CAD locus *SERPINH1* encodes heat-shock protein 47 (Hsp47) (33), a molecular chaperone involved in the collagen secretion pathway. *FN1* encodes fibronectin, a glycoprotein with established roles in cell adhesion, migration, growth, and differentiation. Though increased in atherosclerotic regions, the role of fibronectin in development of CAD remains unclear, with postulated roles in atherogenic lipoprotein retention, direct adverse effects on endothelial cell function, or roles in plaque stability (34). The *TNS1* gene encodes for the tensin-1 protein that attaches the plasma membrane to the extracellular matrix and positively regulates the small Rho GTPase, RhoA (35). The *RHOA* gene itself was identified as a genome-wide significant locus in the latest CARDIoGRAMplusC4D meta-analysis (15) and is predicted to interact with several other CAD genes/pathways in smooth muscle cells and endothelial cells including TGFβ/SMAD3 and ECM proteins, such as collagens and fibronectin (36). RhoA also cooperates with Rac1 and cadherin to regulate barrier function in mural and endothelial cells (37). RhoA activation coincides with endothelial cell inflammation, permeability, and disturbed flow as a result of reduced PPAP2B (itself associated with CAD and ischemic stroke) (38). Lastly, the *ADAMTS7* gene, encoding a metalloproteinase, is proatherogenic based on mouse studies, with a direction of effect consistent with the human genetic association data (39). In the context of its association with CAD, it has been proposed that ADAMTS7 alters smooth muscle cell migration and extracellular matrix composition (40).

### Inflammation and immune pathways

The role of inflammation in CAD pathogenesis is now well-established, yet the number of inflammatory genes mapping to CAD-associated loci is under-represented. One of the main CAD loci involved in inflammation is the interleukin 6 receptor (*IL6R*), which binds the pro-inflammatory cytokine IL-6 and its pathways have been causally linked to CAD using Mendelian randomization analyses (41). Another example is the CAD-associated *CXCL12* gene, which encodes an anti-inflammatory cytokine (also known as stromal derived factor 1; SDF-1) that binds the chemokine (C-X-C motif) receptor CXCR4, a G-protein coupled receptor. Given that *CXCL12* is induced immediately after vessel injury and specifically expressed in atherosclerotic lesions, this gene has potential to serve as a biomarker for early detection (42). The CAD-associated *SH2B3* gene encoding an adapter protein known as LNK is involved in hematopoiesis and suppression of cytokines and thrombopoietin signaling (43). In mice, loss of Sh2b3 was shown to promote both atherosclerosis and thrombosis only under the setting of hypercholesterolemia, suggesting an involvement in platelet/leukocyte activation during atherogenesis (44). It may also serve as an inflammatory link between vascular endothelial cells and immune cells and therapeutic target for hypertension and end-organ inflammation (45). Finally, the ligand *VEGFA* and the VEGF receptor (*FLT1*) loci both associate with CAD; inflammatory conditions in the plaque promote the release of angiogenic factors that result in neovascularization, plaque remodeling, and plaque instability (46).

## Current therapies for CAD

Current therapies for CAD primarily focus on alleviating the symptoms of ischemic events as well as preventing thrombosis from ruptured plaque. Here we review the current treatments for CAD and also provide a genetically informed perspective on these drug targets (Table 1).

**Table 1:** List of current target genes for management of coronary artery disease and their genetic associations.

**Target (gene name)**	**Genetic association with CAD/lipid trait/BP**	**Associated trait (lead SNP)**	**Drug(s)**	**Role**	**Phase**	**Administration**
HMG-Coenzyme A reductase (*HMGCR*)	Yes	LDL-C (rs7703051) (47, 48) Total cholesterol (rs10038095) (49) Plasma LDL-C response to simvastatin (rs17244841) (50) Lipid traits (rs10045497) (51)	Statins (various)	Pharmacological inhibitors	Available with prescription	Oral
Cholesterol absorption in small intestine (gene unclear)	NA	NA	Ezetimibe (Zetia, Ezetrol)	Pharmacological inhibitor	Available with prescription	Oral
ATP citrate lyase (*ACLY*)	NA	NA	Bempedoic acid (Esperion)	Pharmacological inhibitor	Clinical trials (phase 3)	Oral
ApoB-100 (*APOB*)	Yes	LDL-C (rs1367117) (52–54) Total cholesterol (rs1367117) (52–54) Lipid metabolism phenotypes (rs1367117) (55) Triglycerides (rs1042034) (53) HDL-C (rs1042034) (53) Oxidized LDL (rs676210) (56) Lipid metabolism phenotypes (rs676210) (55) LDL-C (rs693) (47, 57–59) Total cholesterol (rs693) (57) Triglycerides (rs693) (48)	Mipomersen (Kynamro)	Antisense oligonucleotide (targets mRNA)	Available with prescription	Injection
PCSK9 (*PCSK9*)	Yes	CAD (rs11591147) (15, 20) LDL-C (rs11591147) (48, 49)	Repatha (evalocumab)	Monoclonal antibody	Available with prescription	Injection
			Praluent (alirocumab)	Monoclonal antibody	Available with prescription	Injection
			Bococizumab	Monoclonal antibody	Clinical trials	Injection
			Inclisiran	Long acting small interfering RNA (siRNA)	Clinical trials (phase 3)	Injection
Lipoprotein A (Lp(a)) (*LPA*)	Yes	CAD (rs10455872) (15) CAD (rs186696265) (20) LDL-C in response to statins (rs10455872) (60)	AKCEA-Apo(a)-LRx	Antisense oligonucleotide	Clinical trials (phase 2b)	Injection
ApoCIII (*APOC3*)	Yes	Triglyceride levels (rs76353203) (80) HDL-C (rs76353203) (80)	AKCEA-ApoCIII-LRx	Antisense oligonucleotide	Clinical trials (phase 2b)	Injection
ANGPTL3 (*ANGPTL3*)	Yes	Triglyceride levels (rs2131925) (52, 53) LDL-C (rs2131925) (52, 53) Total cholesterol (rs2131925) (52, 53)	AKCEA-ANGPTL3-LRx	Antisense oligonucleotide	Clinical trials (phase 2)	Injection
Interleukin 1 beta (*IL1B*)	NA	NA	Canakinumab	Monoclonal antibody	Clinical trials (phase 3)	Injection
Cyclooxygenase-2 (COX-2) (*PTGS2*)	NA	NA	Acetylsalicylic acid (ASA, Aspirin)	Pharmacological inhibitor (general anti-inflammatory effects)	Commercially available	Oral
P2Y_12_ subunit of ADP receptor (*P2RY12*)	NA	NA	Clopidogrel (Plavix)	Pharmacological inhibitor	Available with prescription	Oral
Angiotensin converting enzyme (ACE) (*ACE*)	Yes	Diastolic blood pressure (rs4308) (61, 62)	ACE inhibitors	Pharmacological inhibitors	Available with prescription	Oral
Beta adrenergic receptor(s) (*ADRB1, ADRB2, ADRB3*)	NA	NA	Beta blockers	Pharmacological inhibitors	Available with prescription	Oral
Rho kinase (*ROCK1, ROCK2*)	NA	CAD, sudden cardiac arrest (rs6716724) (63)	Fasudil	Pharmacological inhibitor	Approved in China and Japan	Oral

### Statins

Statins represent the first line of treatment for elevated LDL-cholesterol levels associated with hyperlipidemia and CAD. By inhibiting HMG-coA (hydroxy-3-methylglutaryl-coenzyme A) reductase, statins decrease the production of cholesterol in the liver, thereby reducing its concentration in the circulation. Statins exhibit a pleiotropic effect by attenuating other risk factors for CAD (64). Genetic studies have identified variations in the *HMGCR* gene (rs12916) consistently associated with both blood lipids and LDL-cholesterol (52, 53), while an intergenic variant near *HMGCR* is also associated with CAD in the combined CARDIoGRAMplusC4D and UK Biobank analysis (20).

### Anti-platelet therapies

As a prophylactic measure against thrombosis, antiplatelet drugs are utilized to reduce the risk of myocardial infarction. Two of the more popular antiplatelet drugs are acetylsalicylic acid (ASA) and clopidogrel. ASA is a COX inhibitor that prevents platelet activation by inhibiting the synthesis of thromboxane A2. On the other hand, clopidogrel is an ADP receptor (P2Y12) antagonist that prevents platelet aggregation and further amplification of the activation signal through the downregulation of glycoprotein IIb/IIIa receptor on its surface (65). While the gene targets of these drugs (*PTSG2* and *P2Y12*) do not harbor variants specifically associated with CAD, some of the effector signaling molecules in the pathway (*RHOA, ITGB5*, and *SH2B3*) indeed have CAD associations, as described above. This may represent an opportunity to understand some of the heterogeneity in responses to these commonly used agents by using a pathway approach.

### ACE inhibitors and beta blockers

Two classes of drugs, angiotensin converting enzymes (ACE) inhibitors and beta blockers both function in the maintenance of normal blood pressure. In the endothelium, ACE catalyzes the conversion of angiotensin I to angiotensin II where the latter is a potent vasoconstrictor. Additionally, ACE upregulation results in the degradation of bradykinin, a vasodilatory factor involved in the upregulation of nitric oxide and prostaglandins (66). Given the numerous CAD associations within the NO/cGMP pathway, the efficacy or toxicity profile of these drugs may be influenced by individual genetic variation. Beta blockers exert their cardioprotective effects by intervening in the adrenergic nervous system as competitive antagonists in both the myocardium and vasculature, depending on their selectivity for beta1 or beta2-adrenergic receptors. Clinically, reduced catecholamine stimulation results in decreased cardiac stress leading to decreased heart rate and blood pressure (67, 68). The third generation of beta blockers were shown to have more potent blood pressure lowering effects. Although it may be reasonable to speculate that NO mediated signaling is involved, it was recently demonstrated that nebivolol (compared to metoprolol) suppresses ET-1 mediated vasoconstriction to lower BP (69). This is important given that variation at the ET-1 gene *EDN1* (rs1629862) and the ET-1 receptor type A gene *EDNRA* (rs6841581) were recently identified as CAD loci (20).

### Anti-inflammatory therapies

Therapies targeting inflammatory pathways have been extensively explored in cardiovascular disease. Two recent studies investigating the role of clonal expansion of hematopoietic cells as a potential driver for age-related onset of atherosclerosis have provided evidence that IL1β secretion from TET2 deficient macrophages plays a role in the acceleration of disease (70, 71). TET2 is an epigenetic modifier that negatively regulates the expression of IL1β. Thus, loss of function of TET2 results in the upregulation of IL1β and IL-6 secretion from lesional macrophages (70). This elevated level of proinflammatory cytokines was positively correlated with increased plaque size in the aorta as well as severity of coronary artery calcification in mice and human patients, respectively(70, 71). Studies such as these underscore the potential of targeting the IL1β pathway in slowing down atherosclerosis progression.

The CANTOS (NCT01327846) clinical trial provided critical evidence that targeting IL1β alone with the monoclonal antibody canakinumab can reduce major cardiovascular events along with proinflammatory cytokines (IL-6) and high sensitivity C reactive protein in patients with atherosclerosis. Although the intermediate dose (150 mg) met the primary endpoint of reducing nonfatal myocardial infarction, nonfatal stroke, or cardiovascular death, a significant risk of fatal infection relative to placebo was observed (72). In addition to the high pricing and safety concerns, the marginal clinical benefits demand more development in this area. Given that IL-6 is a causal risk factor for CAD (73), anti-inflammatory therapies remain an attractive therapeutic approach for patients that do not respond to standard lipid lowering medication.

## New CAD therapies informed from genetic studies

### PCSK9 inhibitors and antisense oligonucleotides

One example of newly approved drug targets that have origins in genetic studies is the development of monoclonal antibodies against PCSK9. PCSK9 is a liver protease that targets LDL receptors for lysosomal degradation. The therapeutic potential of targeting PCSK9 was validated through Mendelian randomization studies that correlated a deleterious mutation in this gene with decreased risk (74). Large clinical trials [e.g., FOURIER (NCT01764633), ODYSSEY (NCT01623115)] demonstrated that inhibition of this enzyme reduced systemic LDL levels to a greater extent than maximum statin therapy, with the most recent ODYSSEY trial (NCT01663402) reporting a reduction in both cardiovascular events and all-cause mortality for the first time. In addition to monoclonal antibodies, antisense oligonucleotides have also been developed against PCSK9, which should be evaluated for clinical outcomes in the near future.

### Lipoprotein A and APOC3 antisense oligonucleotides

A high level of circulating lipoprotein A [Lp(a)] is considered a risk factor for cardiovascular disease. Two SNPs, rs3798220 and rs10455872, located within the lipoprotein A (*LPA*) gene correlate with increased levels of Lp(a) and are associated with increased risk for CAD. As one of the first therapies targeting lipoprotein A, AKCEA-APO(a)-L_Rx_ is an antisense oligonucleotide that binds LPA mRNA leading to its degradation. Phase 2 clinical trial data has suggested that this approach is well tolerated and significantly reduced Lp(a) plasma concentrations (65, 66). Similarly, an antisense therapy was developed targeting *APOC3*, a gene involved in regulating plasma triglyceride levels. The antisense oligonucleotide therapy, volanesorsen was shown to reduce cellular levels of APOC3 and led to an overall reduction of triglyceride levels in phase 3 clinical trials (70, 71).

### RhoA-ROCK inhibition

The RhoA-Rock signaling pathway offers another avenue for CAD therapeutic targets. Aberrant activation of this signaling cascade has been implicated in vasoconstriction and endothelial dysfunction. Given the recent CAD association (rs7623687) at *RHOA*, further investigation is warranted to determine how to specifically target this gene. One opportunity is to target the downstream effectors, Rho-associated protein kinases (ROCK1, ROCK2), which control actin cytoskeleton arrangement, cell migration and contractility (75). In particular, a Rock2 inhibitor, Fasudil, has already been tested in clinical trials as a possible therapeutic for CAD as a vasodilatory agent through the upregulation of nitric oxide. It is also noteworthy to mention that Fasudil has been approved as a treatment option for cerebral vasospasm in Japan and China (75).

## Perspectives and future directions

### Genetic risk scores

Besides the 9p21 locus, most loci uncovered from GWAS of CAD have small effects with odds ratios between 1.05 and 1.30. Nonetheless, GWAS results can be utilized to generate genetic risk scores for individuals based on the number of risk alleles they harbor. Therefore, in addition to traditional drug treatments such as statins, individuals that fall within the high CAD risk range based on their genetic risk score can be selected for more aggressive therapies and/or novel CAD treatments as mentioned above. With more data from sources such as the UK Biobank, the Million Veterans Project, and the NIH-funded All of Us project on the horizon, genetic risk scores will have more clinically-relevant predictive utility (76).

### Feasible vs. difficult drug targets

Since GWAS has highlighted the role of vessel wall genes and signaling pathways in the pathogenesis of CAD, it will be critical to apply this knowledge toward vessel wall therapeutic development. Strategies include non-specific targeting of the vessel wall (through upstream or downstream effector molecules), specifically targeting plaque vasculature, or specific cellular phenotypes (e.g., activated resident macrophages or phenotypically modulated smooth muscle cells).

### Target new cell types (e.g., endothelial cells, smooth muscle cells, macrophages)

A CAD protective variant upstream of *ADAMTS7* confers greater protection against CAD for never-smokers compared to those that have smoked 100 or more cigarettes in their lifetime (77). This example highlights the importance of taking into account environmental factors in managing treatments. Other potential targets include the receptors for endothelin-1 on smooth muscle cells. Many of these potential vessel wall target proteins affect smooth muscle cell proliferation and migration, originally believed to drive atherogenesis. The current view suggests smooth muscle cell proliferation and migration could be reparative and promote plaque stability (78). Once the roles and timing of smooth muscle cell proliferation and migration are clarified, the TGF beta and PDGF pathways may be attractive targets due to their role in the regulation of smooth muscle cell genes.

### Machine learning/systems approaches

While GWAS has uncovered invaluable insights into potential therapies and validated existing ones, these associations require extensive follow-up to pinpoint causal variants, genes, pathways. More advanced algorithms such as machine learning can be leveraged to prioritize targets with diverse data inputs such as electronic health records, clinical notes, and -omics. These approaches can help to systematically decrease noise, reduce features, and identify gene sets of interest in addition to common GWAS methods of odds ratios, *p*-value statistics, and chi-square comparisons. Unsupervised learning algorithms have the capability to provide researchers and clinicians with an unbiased network of candidate genes that account for the greatest *variance* in CAD related phenotypes. A specific example is the use of machine learning for drug repurposing based on finding patterns from multi-dimensional datasets. Specific tools have been developed to provide an out-of-the-box approach for understanding diverse text, biological, and medical record data for non-data scientists. One such tool, RepurposeDB, combines drug and disease information to create a reference database for drug repositioning research (79). With the rapidly growing costs of drug discovery/development, such data-informed approaches can offer significant progress for the field.

## Concluding remarks

In summary, in this brief review we bring attention to the genetic loci discovered over the past decade which play critical roles in the vessel wall. Many of these genes are organized into distinct functional pathways, which will help redefine some of the pathogenic mechanisms and prioritize those pathways for future drug development or repurposing strategies.

## Author contributions

AT and CM conceived of the manuscript. AT, DW, CD, and CM wrote the manuscript.

### Conflict of interest statement

The authors declare that the research was conducted in the absence of any commercial or financial relationships that could be construed as a potential conflict of interest.
